# (De)coding perinatal deaths: applying the International Classification of Diseases to perinatal mortality to a perinatal e-registry from 16 hospitals in Benin, Malawi, Tanzania, and Uganda

**DOI:** 10.1080/16549716.2026.2709890

**Published:** 2026-08-04

**Authors:** María del Rosario Alsina, Aliki Christou, Phillip Wanduru, Bianca Kandeya, Muzdalifat Abeid, Effie Chipeta, Hussein Kidanto, Andrea Barnabas Pembe, Jean-Paul Dossou, Peter Waiswa, Lenka Beňová, Claudia Hanson

**Affiliations:** a Department of Global Public Health, Karolinska Institutet, Stockholm, Sweden; b Sexual and Reproductive Health Unit, Institute of Tropical Medicine, Antwerp, Belgium; c School of Public Health, Makerere University, Kampala, Uganda; d Centre for Reproductive Health, Kamuzu University of Health Sciences, Blantyre, Malawi; e Medical College, Aga Khan University, Dar es Salaam, United Republic of Tanzania; f Department of Obstetrics and Gynecology, Muhimbili University of Health and Allied Sciences, Dar es Salaam, United Republic of Tanzania; g Centre de Recherche en Reproduction Humaine et en Démographie, Cotonou, Benin

**Keywords:** Stillbirth, neonatal death, sub-saharan Africa, cause of death, registry

## Abstract

**Background:**

To reduce perinatal deaths, identification of their causes is paramount. ‘The WHO application of ICD-10 to deaths during the perinatal period’ (ICD-PM) was developed to improve the quality of perinatal death data.

**Objectives:**

To determine stillbirth and very early neonatal death rates across 16 hospitals in Benin, Malawi, Tanzania, and Uganda, examine causes of death and associated maternal conditions applying the ICD-PM, and assess how a clinical perinatal e-registry performed when applying the ICD-PM.

**Methods:**

We used cross-sectional data collected between 1st July 2021 and 29th February 2024 of babies weighing ≥1000 g or ≥28 weeks of gestational age, born to women aged 13–50 years in the participating hospitals.

**Results:**

After analyzing 143,105 births, the stillbirth rate was 36.9 per 1,000 births and very early neonatal death rate was 7.7 per 1,000 live births. Nine in ten antepartum stillbirths could not be assigned a cause of death. Among intrapartum stillbirths, the most common cause of death was ‘disorders of fetal growth’ and for very early neonatal death it was ‘complications of intrapartum events’. The e-registry provided information to report on 17 out of the 24 ICD-PM categories.

**Conclusions:**

Collecting high-quality clinical data through an e-registry allowed the identification of a cause of death for most intrapartum stillbirths and very early neonatal deaths. A specifically designed clinical questionnaire and simple diagnostic procedures could enable the application of the ICD-PM in settings with limited diagnostic resources and high mortality rates.

## Paper Context

- **Main findings:** After applying the International Classification of Diseases to Perinatal Mortality classification system to a clinical dataset, a cause of death or an associated maternal condition was established for the majority of perinatal deaths, while most intrapartum stillbirths were a consequence of disorders of fetal growth, complications of intrapartum events were the main cause of very early neonatal deaths.
- **Added knowledge:** This study reveals that collecting basic clinical information through a structured questionnaire in high-burden and resource-limited settings can support the application of the International Classification of Diseases to Perinatal Mortality.
- **Global health impact for policy and action:** This study highlights that despite revisions and updates are needed, the International Classification of Diseases to Perinatal Mortality is still a valuable instrument that could help better understand the pathways to perinatal death, thus contributing to the development of targeted prevention strategies.

## Background

Reducing perinatal deaths is a global health priority to reach the target of less than 12 deaths per 1,000 births by 2030 [1,2]. Each year, 1.9 million babies die before birth and 2.4 million die during the first 28 days of life globally [3,4]. This burden is unequally distributed; sub-Saharan Africa (SSA) has the highest stillbirth rates and newborns are over ten times more likely to die during the first 28 days of life compared to high-income countries [3,5]. Furthermore, this region has shown the least advancements in perinatal mortality reduction over the past two decades [5,6]. A large proportion of these deaths are preventable highlighting the need for targeted interventions to reduce stillbirth and neonatal mortality in SSA [7].

Appropriate identification of risk factors and of causes of perinatal death is crucial to inform strategies to improve survival [7,8]. Two systematic reviews on determinants of stillbirths in SSA found poor data quality resulting from a lack of standardized definitions and other methodological limitations [9,10]. Furthermore, these studies did not report findings by timing of stillbirth, which is relevant given that antepartum and intrapartum stillbirths have different underlying causes [9]. These data gaps prevent from accurately identifying and targeting causes of stillbirth and early neonatal deaths [9].

Perinatal death classification systems were developed to group causes of death based on timing and underlying pathways, and standardize reporting, allowing comparisons within and between countries, and identification of trends and changes across time [11]. ‘The WHO application of ICD-10 to deaths during the perinatal period’ (ICD-PM) classification system was developed to support consistent perinatal death data collection [12]. The ICD-PM applies codes from the International Classification of Diseases, 10^th^ edition (ICD-10) to deaths based on timing of death (antepartum, intrapartum, or neonatal periods) [12]. Each of these three time-of-death groups encompasses a specific number of cause-of-death categories, which are further divided into subcategories corresponding to ICD-10 codes.

The ICD-PM has several advantages over the more than 80 existing classification systems [13,14]. First, it has a multilayered structure to allow the use of data with varying levels of detail which facilitates its application in settings with a wide range of diagnostic resources and was pilot tested with data from high- and middle-income settings [15–19]. Second, it links maternal conditions to perinatal deaths to improve understanding of the underlying processes of perinatal deaths [1,12]. Finally, studies applying the ICD-PM in high-income settings reported high inter-coder agreement rates and reductions in proportion of unexplained stillbirths [18,20].

Although the ICD-PM was intended for use in diverse contexts, accurate classification of perinatal deaths is still highly context-dependent. In a study of over 1,200 stillbirths across four SSA countries, cause of death remained unspecified for over 80% of stillbirths after applying the ICD-PM [16]. A study from China suggested that fetal and maternal clinical data coupled with placental histopathology are sufficient to explain most stillbirths [20]. However, there are currently no recommendations about the amount of data required to ensure the effective application of the ICD-PM in high-burden and limited-resource settings. Minimum data requirements are needed to improve documentation and develop targeted interventions to prevent stillbirths and neonatal deaths [11].

Data on stillbirths and neonatal deaths is urgently needed in SSA to guide efforts to reduce the unacceptably high mortality rates. To support policies and programming of data systems for improved classification of causes of death, we aimed to i) determine stillbirth and very early neonatal death rates across 16 high-caseload hospitals in Benin, Malawi, Tanzania, and Uganda, ii) examine the distribution of main causes of death and associated maternal conditions by time of death applying the ICD-PM, and iii) assess to what extent data from a clinical perinatal e-registry from 16 hospitals with limited diagnostic resources includes the information required to apply the ICD-PM.

## Methods

### Study design

This was a cross-sectional study using data collected prospectively between 1st July 2021 and 29th February 2024 in a perinatal e-registry established in 16 hospitals in Benin, Malawi, Tanzania, and Uganda as part of the ALERT research project [21,22]. This study was carried out in accordance with the principles of the Declaration of Helsinki. Informed consent from participants was not required as all data collected in the e-registry was de-identified; anonymization procedures were reported elsewhere [22]. Ethical approvals were received from local and national ethics committees in all participating countries.

### Study setting

Participating countries were selected based on existing research collaborations and to reflect diversity in terms of health systems characteristics. In each country, the ALERT study included four hospitals of different types (public or private, district or referral). Details of the hospital selection process are available elsewhere [21]. Table 1 shows country-level socio-demographic, health care coverage, and mortality indicators.

**Table 1.** Socio-demographic indicators for Benin, Malawi, Tanzania, and Uganda.

	Benin	Malawi	Tanzania	Uganda
Population^a^	13,712,828	20,931,751	67,438,106	48,582,334
Income level^b^	Lower-middle	Low	Lower-middle	Low
Percentage of births delivered in a health facility (%)^c^	90.6	96.7^d^	81.2	73.4^e^
Stillbirth rate^f^	20.0	16.1	18.3	15.1
Early neonatal mortality rate^g^	28.6	18.7	19.6	18.4

^a^World Bank Group country data (World Bank, 2023); ^b^World Bank Group country classifications by income (World Bank, 2023); ^c^Global Health Observatory data repository (WHO, 2022); ^d^Global Health Observatory data repository (WHO, 2020); ^e^Global Health Observatory data repository (WHO, 2016); ^f^Expressed per 1,000 births (UNICEF. Stillbirths and stillbirth rates [Internet]. UNICEF DATA; 2023); ^g^Expressed per 1,000 live births (UNICEF. Neonatal mortality rate [Internet]. UNICEF Data Warehouse; 2024).

### Study population

All babies born in participating hospitals to women aged between 13 and 50 years, with a birthweight of 1000 g or more or, if birthweight was not available, of a gestational age of 28 weeks or more, were eligible. These criteria reflect the inclusion to the ALERT study; maternal age corresponds to the reproductive age range, and newborn criteria align with international definitions of late gestational stillbirth. Although gestational age was recorded, its measurement was based on last menstrual period, therefore, due to potential measurement error, birthweight was used as the primary inclusion criterion. All eligible babies were included if they were reported as stillborn at birth or if they were born alive and had a reported outcome within the first 24 h after birth. We restricted the analysis to 24 h after birth given that deaths occurring after discharge and before 7 days of life could not be captured. No formal sample calculation was performed.

### Data sources and data collection

Data was collected by maternity staff or data clerks who first identified eligible women, assigned them a unique identifier, and then reviewed maternal antenatal cards, hospital admission books, labor records, and delivery and postnatal registers. Perinatal health and care indicators were entered on site using the Research Electronic Data Capture platform [21]. To ensure consistency in data collection across countries, an operational manual was developed, and the Research Electronic Data Capture data quality module was set up to assure completeness of key variables [22]. Data quality procedures were implemented at three levels: daily completeness checks by data controllers, weekly consistency and completeness checks by country data managers, and weekly international coordination meetings to monitor data collection and to mitigate any arising challenge. Finally, e-registry data was assessed against routinely collected district-level data, showing high concordance. The e-registry data collection tool was developed based on registries from high-income countries and with input from local experts on our team. The questionnaire comprised 51 questions and collected data on antenatal, labor and birth, and postnatal care (Supplementary table S1). Antenatal indicators included obstetric history and presence of complications during the index pregnancy including, but not limited to high blood pressure, diabetes or infections like Malaria. The need and indication of induction or augmentation of labor or operative birth as well as fetal characteristics, like birthweight, sex, and Apgar score, were captured among other intrapartum care indicators. Finally, postnatal care indicators comprised maternal complications like postpartum hemorrhage, perinatal morbidity, like seizures or neonatal infection, and interventions performed. In the case of multiple pregnancy, data on each baby were collected separately under the same maternal identification number.

### Determination of time of death

We adopted the 11th International Classification of Diseases’ definition of late gestation stillbirth as the death of a baby of a birthweight of 1000 g or more or of a gestational age of 28 weeks or more before or during labor [23]. Timing of stillbirth was based on fetal appearance; signs of skin maceration were considered an indication that death occurred over 12 h before the onset of labor and were used as a surrogate for antepartum stillbirths [24]. The absence of maceration was considered a proxy for intrapartum stillbirth. To assess the robustness of this classification, we conducted a sensitivity analysis including only those stillbirths who had a recorded fetal heartrate at admission. We defined very early neonatal death as the death of a live-born baby during the first 24 h of life [23]. A composite binary variable was created to report very early neonatal death if baby status was reported as dead within the first 24 h in our e-registry.

### Application of the ICD-PM

After determining time of death, we reviewed our questionnaire to identify variables that best represented the ICD-10 codes for each cause of death category. If no variables in our dataset captured information relevant to a specific code, we searched for variables that reflected the category’s description. For example, our dataset captured clinical information on neonatal infection, however, etiological diagnosis was not available. Therefore, our variable represented the category ‘A2: Infection’ although it did not match any of the ICD-10 codes within this category. We conducted the same process for the five categories of associated maternal conditions.

**Figure 1.**
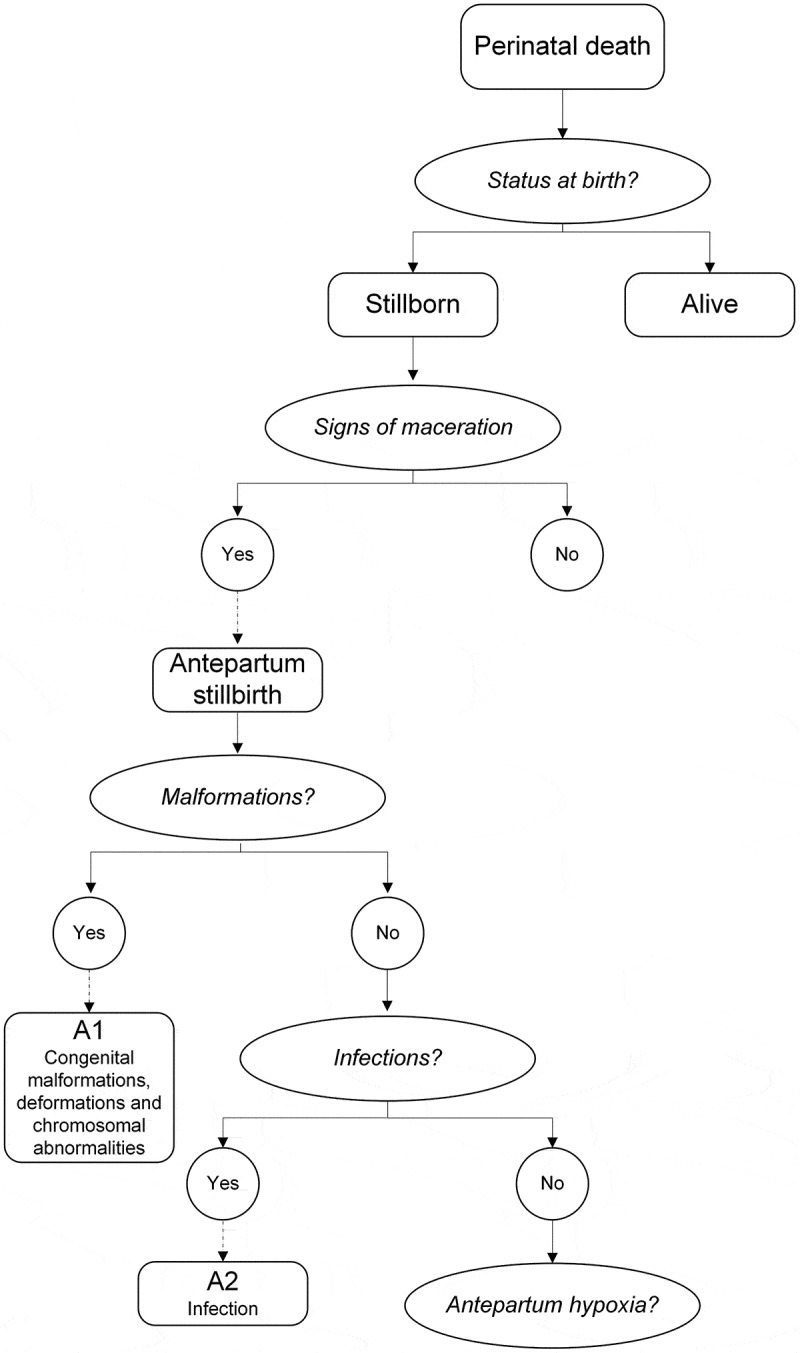
ICD-PM application algorithm.

To examine causes of death, we created composite variables to reflect each ICD-PM classification category following the ICD-PM guidelines [12]. A total of 22 variables from the ALERT e-registry were used to create composite variables for cause of death categories. To create variables representing maternal condition categories, we combined 37 ALERT e-registry variables. The application process was conducted following a rule-based algorithm. For each death, categories were applied sequentially in a fixed order and based on available data, rather than through a case-by-case review. For example, if a case met the criteria for category A1, it was assigned to that category, and no further categories were considered. If criteria were not met, the case was evaluated against the next category in the hierarchy, in this case A2, and this process continued until a cause of death was assigned or all categories had been exhausted. A diagram illustrating the classification process is provided in Figure 1. The process of operationalizing and implementing the classification was conducted by a pediatrician with clinical and research experience (MRA), in consultation with three senior researchers; a specialist in obstetrics and gynecology with a PhD in epidemiology, an expert in population health with vast experience in maternal and child health, and a public health researcher with expertise in maternal, perinatal, and newborn health (CH, LB, AC).

To assess the extent to which our data covered the ICD-PM codes and categories, we created four groups. For each cause of death or maternal condition category, we assessed if our data covered all the ICD-10 codes within a category (detailed data), some codes (partially detailed data), only the main category without sufficient information to assign a specific ICD-10 code (not detailed data) or neither the category nor codes (no data). We labelled categories where no specific data was required (i.e. ‘antepartum unspecified cause of death’) as ‘not applicable’. The application of the ICD-PM to the ALERT data, including level of data detail for each ICD-PM category and the ALERT variables used to represent them, is presented as supplementary material (Supplementary table S2).

### Analysis

We analyzed pooled data from 16 hospitals using Stata 17 (StataCorp, 2021, Stata Statistical Software: Release 17, College Station, TX: StataCorp LLC.). Main maternal, obstetric, and fetal characteristics were selected based on previous literature and clinical experience to include those which are associated with perinatal death and were available in our dataset. Categorical variables were created to report maternal age, parity at index pregnancy, gestational age and birthweight, category boundaries are shown in Table 2. Missing values for each variable were coded as a separate category. Stillbirth and very early neonatal death rates were reported per 1,000 births and 1,000 live births, respectively [25]. We used descriptive statistics to report cause of death and associated maternal conditions as frequencies and percentages; no outcomes, exposures, predictors, potential confounders, or effect modifiers were needed for the analysis. The assessment of the e-registry data was also descriptive, and results are reported as frequencies of ICD‑PM categories by level of data detail.

**Table 2.** Main maternal, obstetric, and fetal characteristics of 6,338 perinatal deaths across 16 hospitals in Benin, Malawi, Tanzania, and Uganda.

	Benin *n* = 2,439		Malawi *n* = 1,320		Tanzania *n* = 465		Uganda *n* = 2,114		Pooled *n* = 6,338	
	n	%	n	%	n	%	n	%	n	%
Maternal age (years)										
<20	226	9.3	378	28.6	67	14.4	358	16.9	1,029	16.2
20–29	1,192	48.9	604	45.8	209	44.9	1,144	54.1	3,149	49.7
30–39	920	37.7	299	22.7	144	31.0	570	27.0	1,933	30.5
≥40	101	4.1	35	2.7	45	9.7	42	2.0	223	3.5
Missing	0	0.0	4	0.3	0	0.0	0	0.0	4	0.1
Parity at index pregnancy										
0	569	23.3	599	45.4	149	32.0	688	32.5	2,005	31.6
1–3 births	1,184	48.5	574	43.5	249	53.5	952	45.0	2,959	46.7
≥4 births	652	26.7	143	10.8	63	13.5	470	22.2	1,328	21.0
Missing	34	1.4	4	0.3	4	0.9	4	0.2	46	0.7
Poor outcome in immediate previous pregnancy										
No	1,545	63.3	629	47.7	266	57.2	1,159	54.8	3,599	56.8
Yes^a^	344	14.1	128	9.7	64	13.8	249	11.8	785	12.4
No previous pregnancy	513	21.0	558	42.3	133	28.6	621	29.4	1,825	28.8
Missing	37	1.5	5	0.4	2	0.4	85	4.0	129	2.0
Number of antenatal care visits for index pregnancy										
None	21	0.9	86	6.5	1	0.2	22	1.0	130	2.1
1–3	1,033	42.4	696	52.7	75	16.1	1,149	54.4	2,953	46.6
4–7	1,296	53.1	508	38.5	370	79.6	881	41.7	3,055	48.2
≥8	66	2.7	12	0.9	19	4.1	26	1.2	123	1.9
Missing	23	0.9	18	1.4	0	0.0	36	1.7	77	1.2
Antenatal complications in index pregnancy^b^	1,154	47.4	240	18.2	172	37.1	600	28.5	2,166	34.3
Multiple pregnancy	240	9.8	91	6.9	45	9.7	185	8.8	561	8.9
Fetal heartbeat at admission										
Positive	806	33.0	653	49.5	142	30.5	494	23.4	2,095	33.1
Negative	1,462	59.9	547	41.4	276	59.4	1,187	56.1	3,472	54.8
No information	171	7.0	120	9.1	47	10.1	433	20.5	771	12.2
Referred from another health facility	1,941	79.6	261	19.8	85	18.3	661	31.3	2,948	46.5
Mode of birth										
Vaginal	1,562	64.0	930	70.5	282	60.6	1,308	61.9	4,082	64.4
Cesarean Section	836	34.3	305	23.1	163	35.1	765	36.2	2,069	32.6
Assisted breech	21	0.9	60	4.5	12	2.6	40	1.9	133	2.1
Operative vaginal	20	0.8	25	1.9	8	1.7	1	0.0	54	0.9
Gestational age (weeks)										
<32	339	13.9	108	8.2	35	7.5	193	9.1	675	10.7
32–36	701	28.7	273	20.7	152	32.7	467	22.1	1,593	25.1
37–41	1,283	52.6	890	67.4	257	55.3	1,185	56.1	3,615	57.0
≥42	78	3.2	22	1.7	13	2.8	76	3.6	189	3.0
Missing	38	1.6	27	2.0	8	1.7	193	9.1	266	4.2
Birthweight (grams)										
<1500	279	11.4	148	11.2	69	14.8	211	10.0	707	11.2
1500–2499	811	33.3	418	31.7	175	37.6	529	25.0	1,933	30.5
2500–3999	1,304	53.5	706	53.5	208	44.7	1,221	57.8	3,439	54.3
≥4000	36	1.5	23	1.7	11	2.4	114	5.4	184	2.9
Missing	9	0.4	25	1.9	2	0.4	39	1.8	75	1.2
Baby’s sex										
Female	1,163	47.7	579	43.9	220	47.3	946	44.7	2,908	45.9
Male	1,273	52.2	735	55.7	242	52.0	1,164	55.1	3,414	53.9
Missing	3	0.1	6	0.5	3	0.6	4	0.2	16	0.3

^a^Includes miscarriage, stillbirth, and neonatal death

^b^Includes: hypertensive disorder, preterm labor, pre-labor rupture of membranes, cardiac/renal diseases, gestational diabetes, diabetes mellitus, malaria, and severe anemia

## Results

**Figure 2.**
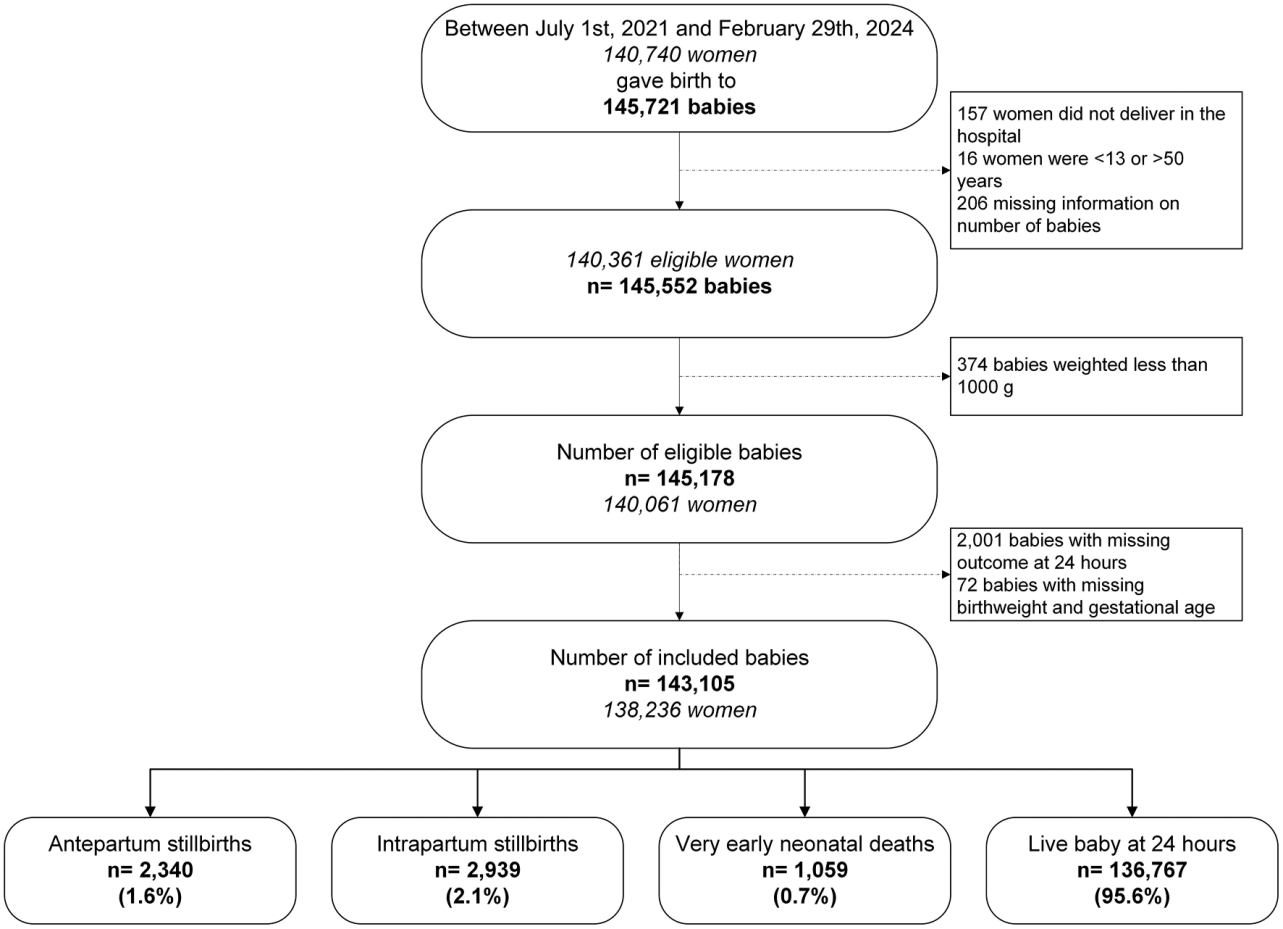
Participant inclusion flowchart.

During the study period, 145,721 babies were born to 140,740 women (Figure 2). We included 143,105 babies from 138,236 mothers in the analysis: 2,340 (1.6%) antepartum stillbirths, 2,939 (2.1%) intrapartum stillbirths, 1,059 (0.7%) very early neonatal deaths, and 136,767 (95.6%) alive after 24 h.

### Sample characteristics

In our sample of 143,105 babies, over half of the mothers were between 20–29 years of age (Supplementary table S3). Almost 40% of babies’ mothers were primiparas and nearly 50% had between one and three previous births. Less than 10% had a poor outcome in the immediate previous pregnancy. In 60.5% of our sample of babies, mothers attended between four and seven antenatal care visits and less than 15% were diagnosed with complications. Almost one in five mothers were referred and nearly 30% had a caesarean section delivery. Babies born term and of a normal birthweight represented about 80% of our sample.

### Characteristics of perinatal deaths

Among the 6,338 stillbirths and very early neonatal deaths, most mothers were multiparas (1–3 previous births) and aged 20–29 years (Table 2). Less than 15% had a poor outcome in the immediate previous pregnancy, and most mothers had at least one antenatal care visit during the index pregnancy. Frequency of antenatal complications was close to 35% and referral was 46.5%. Mode of birth was vaginal in over 60% of the sample, followed by caesarean section (32.6%). Over half of the babies were born at term and with a normal birthweight.

### Stillbirth and very early neonatal death rates

The stillbirth rate in our sample was 36.9 per 1,000 births and very early neonatal death rate was 7.7 per 1,000 live births (Table 3). Intrapartum stillbirths represented 55.7% of all stillbirths. The sensitivity analysis excluding births without recorded fetal heart rate at admission showed a contrasting distribution, with antepartum stillbirths representing 72.9% of all stillbirths (Supplementary table S4).

**Table 3.** Stillbirth and very early neonatal mortality rates from 16 hospitals in Benin, Malawi, Tanzania, and Uganda, by country and pooled sample.

	Benin	Malawi	Tanzania	Uganda	Pooled
Number of births	31,933	50,541	23,267	37,364	143,105
Stillbirth rate (per 1,000 births)	68.3	19.3	15.5	47.2	36.9
Range across hospitals	30.7–85.6	12.4–23.9	7.3–31.8	35.5–72.8	
Antepartum stillbirths	42.6%	51.1%	56.5%	40.2%	44.3%
Intrapartum stillbirths	57.4%	48.9%	43.5%	59.8%	55.7%
Very early neonatal death rate (per 1,000 live births)	8.7	7.0	4.5	9.9	7.7
Range across hospitals	3.5–12.5	3.8–9.6	2.2–6.2	6.4–14.0	

The highest stillbirth rates were in Benin, ranging from 30.7 to 85.6 per 1,000 births across hospitals. Very early neonatal death rates were highest in hospitals in Uganda (9.9 per 1,000 live births) followed by hospitals in Benin (8.7). Tanzanian hospitals showed the lowest rates; 15.5 per 1,000 births and 4.5 per 1,000 live births. Rates by hospital are presented as supplementary material (Supplementary table S5).

### Causes of death and associated maternal conditions

#### Antepartum stillbirths

Among antepartum stillbirths, pooled and country-level data showed a similar distribution of causes of death (Table 4 and Supplementary table S6). ‘Unspecified cause of death’ was the most frequent (90.4%), followed by ‘disorders of fetal growth’ (4.9%), and ‘hypoxia’ (2.6%). The highest percentage of unspecified antepartum stillbirths was observed in Malawi (94.0%) and the lowest in Benin (87.7%). We did not identify any associated maternal conditions in 23.8% of antepartum stillbirths. Among the 2,115 antepartum stillbirths of unspecified cause, 75.0% (*n* = 1,596) were associated to a maternal condition of either ‘complication of placenta, cord, and membranes’ (M1, *n* = 165), ‘complication of pregnancy’ (M2, *n* = 324), ‘complications of labor and delivery’ (M3, *n* = 512), or ‘maternal medical or surgical conditions’ (M4, *n* = 595).

**Table 4.** Classification of 6,338 stillbirths and very early neonatal deaths according to cause of death and associated maternal condition using the International classification of diseases to perinatal mortality.

	Maternal conditions*					
	M1	M2	M3	M4	M5	Total (%)
Antepartum death						
A1: Congenital malformations, deformations and chromosomal abnormalities	2	8	12	6	4	32 (1.4)
A2: Infection	3	7	5	3	0	18 (0.8)
A3: Antepartum hypoxia	10	16	22	6	6	60 (2.5)
A4: Other specified antepartum disorders	ND	ND	ND	ND	ND	ND
A5: Disorders related to fetal growth	11	7	31	37	29	115 (4.9)
A6: Fetal death of unspecified cause	165	324	512	595	519	2,115 (90.4)
Total (%)	191 (8.2)	362 (15.5)	582 (24.9)	647 (27.6)	558 (23.8)	2,340 (100.0)
Intrapartum death						
I1: Congenital malformations, deformations and chromosomal abnormalities	4	13	29	15	10	71 (2.4)
I2: Birth trauma	ND	ND	ND	ND	ND	ND
I3: Acute intrapartum event	83	94	316	71	100	664 (22.6)
I4: Infection	0	3	0	1	0	4 (0.1)
I5: Other specified antepartum disorders	ND	ND	ND	ND	ND	ND
I6: Disorders related to fetal growth	299	205	283	220	111	1,118 (38.1)
I7: Intrapartum death of unspecified cause	193	129	427	181	152	1,082 (36.8)
Total (%)	579 (19.7)	444 (15.1)	1,055 (35.9)	488 (16.6)	373 (12.7)	2,939 (100.0)
Very early neonatal death						
N1: Congenital malformations, deformations and chromosomal abnormalities	3	9	22	3	10	47 (4.5)
N2: Disorders related to fetal growth	3	6	25	10	15	59 (5.7)
N3: Birth trauma	ND	ND	ND	ND	ND	ND
N4: Complications of intrapartum events	75	120	295	108	269	867 (83.5)
N5: Convulsions and disorders of cerebral status	0	1	2	1	0	4 (0.4)
N6: Infection	0	0	0	0	0	0
N7: Respiratory and cardiovascular disorders	0	0	0	0	0	0
N8: Other neonatal conditions	ND	ND	ND	ND	ND	ND
N9: Low birth weight and prematurity	5	34	6	5	11	61 (5.9)
N10: Miscellaneous	ND	ND	ND	ND	ND	ND
N11: Neonatal death of unspecified cause	ND	ND	ND	ND	ND	ND
Total (%)	86 (8.3)	170 (16.4)	350 (33.7)	127 (12.2)	305 (29.4)	1,038 (100.0)

*M1: Complications of placenta, cord and membranes; M2: Maternal complications of pregnancy; M3: Other complications of labor and delivery; M4: Maternal medical and surgical conditions; M5: No maternal condition.

ND (no data): no suitable variable was available in the ALERT e-registry.

#### Intrapartum stillbirths

Overall, ‘growth disorders’ (38.0%), ‘unspecified cause of death’ (36.8%), and ‘acute intrapartum events’ (22.6%) were the leading causes of intrapartum stillbirths (Table 4). This was also the distribution in Benin, while in Tanzania ‘acute intrapartum event’ (36.3%) was the second most common and ‘unspecified cause of death’ (20.4%) the third. In Malawi, ‘acute intrapartum events’ (41.7%) was the most frequent and in Uganda it was ‘unspecified cause of death’ (41.8%). ‘Complications of labor and delivery’ (M3) was the most common associated maternal condition overall (35.9%). Among intrapartum stillbirths due to growth disorders, ‘complications of placenta, cord and membranes’ (M1) was the most frequently associated maternal condition (*n* = 299).

#### Very early neonatal deaths

Of the 1,059 very early neonatal deaths, we could classify 1,038 (96.8%) (Table 4). Most were classified as ‘complications of intrapartum events’ (83.5%) followed by ‘low birthweight and prematurity’ (5.9%) and ‘disorders of fetal growth’ (5.7%). Among deaths due to ‘complications of intrapartum events’, the most frequently associated maternal condition was ‘other complications of labor and delivery’ (M3, *n* = 295).

#### Application of ICD-PM

Data collected in the e-registry allowed us to construct 17 cause-of-death categories among the 24 included in the ICD-PM (Supplementary table S2). Excluding antepartum and intrapartum death of unspecified cause, which do not require additional information, our dataset covered over 75% of the data required to apply the ICD-PM with varying levels of detail. We had partially detailed data for eight cause-of-death categories and not detailed data for six. We had no data for seven categories, one in the antepartum death group, two in the intrapartum death group and four in the neonatal death group. We had partially detailed data for all maternal conditions.

## Discussion

This cross-sectional analysis of prospective data collected over 32 months in a perinatal e-registry of 143,105 in-facility births, including 6,338 stillbirths and very early neonatal deaths, from 16 hospitals across Benin, Malawi, Tanzania, and Uganda found an overall stillbirth rate of 36.9 per 1,000 births and a very early neonatal death rate of 7.7 per 1,000 live births. Our clinical dataset performed well in identifying a cause of death among very early neonatal deaths (96.8%), followed by intrapartum deaths (63.2%), while only one in ten antepartum stillbirths could be matched with a specific cause of death. Among intrapartum stillbirths, almost 40% were due to disorders of fetal growth. Over 80% of very early neonatal deaths were due to complications of intrapartum events. Complications of labor and delivery were among the top two maternal conditions across the three outcomes. This e-registry, which was feasible to implement with a high degree of fidelity as part of a large trial, provided sufficient information to report on most ICD-PM categories.

Consistent with earlier research applying the ICD-PM to hospital data from SSA, most antepartum deaths in our study were categorized as unspecified. A study in Kenya, Malawi, Sierra Leone, and Zimbabwe could not assign cause of death in almost 90% of antepartum stillbirths [16]. Furthermore, studies in Zambia and Zanzibar reported unspecified cause in over 70% and in 50% of antepartum deaths, respectively [26,27]. Although these findings could be explained by lack of diagnostic tools to identify conditions such as chromosomal abnormalities or hematological diseases in settings with limited resources, it should be noted that establishing a cause for antepartum stillbirth is challenging even in high-income countries [28,29]. Among antepartum deaths which could be categorized, infections were the most common cause of death in the multi-country study and acute intrapartum events in Zanzibar [16,26]. In contrast, in our study it was disorders related to fetal growth, which could be attributed to the presence of maternal complications, such as hypertension or anemia, in over a third of perinatal deaths.

Most intrapartum stillbirths and very early neonatal deaths in our study had an identified cause. Furthermore, most were due to preventable causes, as reported in a perinatal death audit study in Tanzania [30]. Our finding that the main cause of intrapartum stillbirths was disorders related to fetal growth contrasts with findings from similar settings that report intrapartum events as the main reason. This could be partially explained by our lack of indicators for ‘acute intrapartum events’, such as fetal acidosis or meconium in amniotic fluid [16,26,27]. This interpretation is also supported by our finding that complications of intrapartum events were the most common cause of death among neonates, similar to studies applying the ICD-PM in Zanzibar and Zambia [26,27]. Although collecting information on fetal acidosis could entail a significant investment for settings with limited funding, including characteristics of amniotic fluid in our questionnaire would not have taken additional resources and could have increased the identification of acute intrapartum events. Furthermore, lactate rapid tests are an available and relatively affordable tool [31].

Between 70% and 90% of the deaths in our study were associated with a maternal condition, similar to previous research from SSA [16]. Antepartum stillbirths were most frequently associated with maternal medical conditions, as reported in studies from Nigeria and Zanzibar [26,32]. This finding suggests that a thorough maternal medical history assessing pre-existing diseases, could further improve the detection of associated maternal conditions and, thus, improve the understanding of the causal pathways to stillbirth and neonatal death [28]. Complications of labor and delivery such as malpresentation, malposition and operative birth, were the most frequent among intrapartum stillbirths and very early neonatal deaths. This finding is supported by a global systematic review and meta-analysis of ICD-PM reports and previous research in Zambia [27,33]. Furthermore, it reinforces our speculations that ‘acute intrapartum events’ could be underrepresented in our sample due to lack of clinical indicators of fetal distress.

Our dataset captured enough information to report on most ICD-PM categories despite not being specifically designed for that purpose. By including only a few additional questions in our questionnaire, such as signs of birth injury, or presence of meconium or signs of infection in the amniotic fluid, we would have been able to represent all ICD-PM categories. Like other studies describing the process of applying the ICD-PM to hospital data, we also found that lack of clinical information and limited post-mortem investigations were the main challenges [15,26,34]. While laboratory and imaging examinations are needed to more accurately apply the ICD-PM, there is not enough evidence to recommend an effective diagnostic algorithm [20,26,35]. Nevertheless, recent publications suggest that clinical information coupled with placental histopathology and fetal autopsy are the most useful information to assign a cause of death [36]. Since funding and resources for conducting these examinations might not be available in low- and middle-income countries, external examination of the placenta and the fetus could be an acceptable substitute [36,37]. Therefore, collecting comprehensive clinical data, including characteristics of the placenta, through a standardized questionnaire could be a feasible option. Furthermore, this could be a more efficient way to capture data on cause of death compared to perinatal death reviews given that adequate implementation of facility-based audits in SSA has been poor due to financial, institutional, and cultural factors [38,39]. Moreover, our algorithmic approach to the application of the ICD-PM yielded comparable findings to those of studies from similar settings using audit data, making it a suitable alternative [16,26,27,30,32]. While electronic registers, like the one used in this study, are preferred for this task, infrastructural and human resources issues, including challenges in integrating digitalization into daily tasks, could hinder widespread implementation [40,41]. Alternatively, smart paper technologies, which can be transformed into electronic data through scanning of pre-defined forms, could be a practical solution as they have shown to be accepted by health providers and not increase their workload [42].

While our results indicate that improvements in antenatal and intrapartum care are required to reduce perinatal deaths, the current categories in the ICD-PM do not suffice to develop specific recommendations on which aspects of care should be strengthened [33,43]. Intrapartum events can be a consequence of poor labor management, but they can also be explained by late referrals due to reduced number of ambulances or fuel crises [26,34]. By failing to capture structural factors that are central to understanding perinatal outcomes in our setting, the current version of the ICD-PM does not provide a full picture of the causal pathways to perinatal death in resource-limited settings. Furthermore, the ICD-PM has been criticized for not supporting the identification of potential solutions despite having a great number of categories [44,45]. We therefore suggest that upcoming revisions of the ICD-PM focus on creating a simpler and more action-oriented classification system.

Despite these concerns, the ICD-PM’s tiered structure allowed us to report on most categories solely with clinical data, and the associated maternal conditions helped explain many deaths in our study, as was also reported in Tanzania [26]. Therefore, we believe that the ICD-PM is a useful tool to gather data to inform the development of targeted interventions to reduce stillbirths and very early neonatal deaths in similar settings.

The main strength of our study is applying the ICD-PM classification system to over 6000 perinatal deaths using high-quality standardized e-registry data from 16 hospitals across four SSA countries [22]. This is an important contribution to identifying causes of perinatal death to accurately target prevention efforts in high-mortality settings. However, there are several limitations that should be considered when interpreting our results. First, determination of stillbirth timing relied only on skin appearance at birth. Although information on fetal heartbeat at admission is preferred to accurately establish time of death, our dataset showed high missingness rates in admission monitoring results and thus were not used in our analysis [25]. Furthermore, fetal monitoring at admission may not accurately represent fetal status at the onset of labor, as delays in access to health facilities are common in these settings and women typically present to the hospital after labor has started [46,47]. This is supported by the results of sensitivity analyses showing a skewed distribution towards antepartum stillbirths, which contradicts previous evidence in the SSA context. Second, we used data collected for another purpose to examine cause of death. Although data extracted from death certificates is preferred, these were unavailable in our study [12,15]. Third, there may have been misclassification between stillbirths and very early neonatal deaths. Nevertheless, analyzing the three time-of-death groups still allows for improved prevention strategies which could outweigh diagnostic inaccuracies [48]. Fourth, our rule-based algorithm was not formally assessed. However, our findings are consistent with those from similar settings using a case-by-case approach. Finally, given that they are based on data from 16 hospitals across four sub-Saharan African countries, we consider our results could be generalizable to other countries in this region. Nevertheless, our findings might not be comparable to those resulting from analysis of data from lower-level facilities or community births.

## Conclusions

Our study showed that collecting high-quality clinical data through a standardized dataset with less than 40 variables is sufficient to apply the ICD-PM in resource-limited settings with high perinatal mortality rates. Although diagnostic tests are required to obtain more detailed and accurate data, basic information is still useful to guide prevention efforts. Furthermore, simple diagnostic procedures could help curtail the proportion of stillbirths of unspecified cause. Future revisions of the ICD-PM should include health system level factors and be more relevant for prevention programming to help reduce perinatal death in SSA. Nevertheless, applying the current ICD-PM in these settings can still support the development and strengthen the implementation of targeted interventions, which are key to reducing stillbirths and very early neonatal deaths.

## Supplementary Material

Supplementary material_Decoding perinatal deaths_clean.docx

STROBE checklist_Decoding perinatal deaths.docx

## Data Availability

The data that support the findings of this study are available from the corresponding author, MRA, upon reasonable request.
