# Supplementary material for: (De)coding perinatal deaths: applying the International Classification of Diseases to perinatal mortality to a perinatal e-registry from 16 hospitals in Benin, Malawi, Tanzania, and Uganda
Source: Glob Health Action. 2026 Aug 4;19(1):2709890. doi: 10.1080/16549716.2026.2709890 (PMC13439779; doi:10.1080/16549716.2026.2709890)
Supplement: Supplementary file 1 [file ZGHA_A_2709890_SM5804.docx]

STROBE Statement—checklist of items that should be included in reports of observational studies

|  | Item No. | Recommendation | Page  No. | Relevant text from manuscript |
| --- | --- | --- | --- | --- |
| **Title and abstract** | 1 | (*a*) Indicate the study’s design with a commonly used term in the title or the abstract | p.2, line 28, Abstract |  |
|  |  | (*b*) Provide in the abstract an informative and balanced summary of what was done and what was found | p.2, Abstract |  |
| Introduction | | | |  |
| Background/rationale | 2 | Explain the scientific background and rationale for the investigation being reported | p.5-6, Background |  |
| Objectives | 3 | State specific objectives, including any prespecified hypotheses | p.7, line 91-96, Background |  |
| Methods | | | |  |
| Study design | 4 | Present key elements of study design early in the paper | p.7, Methods: Study design |  |
| Setting | 5 | Describe the setting, locations, and relevant dates, including periods of recruitment, exposure, follow-up, and data collection | p.7, line 99-101, Methods: Study design & p.7, line 107-110, Methods: Study setting |  |
| Participants | 6 | (*a*) *Cohort study*—Give the eligibility criteria, and the sources and methods of selection of participants. Describe methods of follow-up  *Case-control study*—Give the eligibility criteria, and the sources and methods of case ascertainment and control selection. Give the rationale for the choice of cases and controls  *Cross-sectional study*—Give the eligibility criteria, and the sources and methods of selection of participants | p.8, Methods: Study population |  |
|  |  | (*b*) *Cohort study*—For matched studies, give matching criteria and number of exposed and unexposed  *Case-control study*—For matched studies, give matching criteria and the number of controls per case | Not applicable |  |
| Variables | 7 | Clearly define all outcomes, exposures, predictors, potential confounders, and effect modifiers. Give diagnostic criteria, if applicable | p.11, line 179-180, Methods: Analysis |  |
| Data sources/ measurement | 8* | For each variable of interest, give sources of data and details of methods of assessment (measurement). Describe comparability of assessment methods if there is more than one group | p.8-10, Methods: Data sources and data collection & Determination of time of death & Application of the ICD-PM |  |
| Bias | 9 | Describe any efforts to address potential sources of bias | p.8, Methods: Study population & p.8, line 119-124, Methods: Data sources and data collection |  |
| Study size | 10 | Explain how the study size was arrived at | p.8, line 115-117, Methods: Study population |  |

Continued on next page

| Quantitative variables | 11 | Explain how quantitative variables were handled in the analyses. If applicable, describe which groupings were chosen and why | p.9-10, Methods: Determination of time of death & Application of the ICD-PM |  |
| --- | --- | --- | --- | --- |
| Statistical methods | 12 | (*a*) Describe all statistical methods, including those used to control for confounding | p.10-11, Methods: Analysis |  |
|  |  | (*b*) Describe any methods used to examine subgroups and interactions | Not applicable |  |
|  |  | (*c*) Explain how missing data were addressed | p.11, line 175-176, Methods: Analysis |  |
|  |  | (*d*) *Cohort study*—If applicable, explain how loss to follow-up was addressed  *Case-control study*—If applicable, explain how matching of cases and controls was addressed  *Cross-sectional study*—If applicable, describe analytical methods taking account of sampling strategy | Not applicable |  |
|  |  | (*e*) Describe any sensitivity analyses | Not applicable |  |
| Results | | | | |
| Participants | 13* | (a) Report numbers of individuals at each stage of study—eg numbers potentially eligible, examined for eligibility, confirmed eligible, included in the study, completing follow-up, and analysed | p.11, line 182-185, Results & p.12 Results: Figure 1 |  |
|  |  | (b) Give reasons for non-participation at each stage | p.12, Results: Figure 1 |  |
|  |  | (c) Consider use of a flow diagram | p.12, Results: Figure 1 |  |
| Descriptive data | 14* | (a) Give characteristics of study participants (eg demographic, clinical, social) and information on exposures and potential confounders | p.12, Results: Sample characteristics & p.12-13, Results: Characteristics of perinatal deaths  p.13-14, Results: Table 2 |  |
|  |  | (b) Indicate number of participants with missing data for each variable of interest | p.13-14, Results: Table 2 |  |
|  |  | (c) *Cohort study*—Summarise follow-up time (eg, average and total amount) | Not applicable |  |
| Outcome data | 15* | *Cohort study*—Report numbers of outcome events or summary measures over time | Not applicable |  |
|  |  | *Case-control study—*Report numbers in each exposure category, or summary measures of exposure | Not applicable |  |
|  |  | *Cross-sectional study—*Report numbers of outcome events or summary measures | Numbers and summary measures of stillbirth and very early neonatal death rates are reported in the Results section: p.14, line 209-210, Results: Stillbirth and very early neonatal death rates  p.14, Results: Table 3  Numbers and summary measures of cause of death and associated maternal conditions are reported in the Results section: p.15-16, Results: Cause of death and associated maternal conditions  p.16-17, Results: Table 4 |  |
| Main results | 16 | (*a*) Give unadjusted estimates and, if applicable, confounder-adjusted estimates and their precision (eg, 95% confidence interval). Make clear which confounders were adjusted for and why they were included | Not applicable |  |
|  |  | (*b*) Report category boundaries when continuous variables were categorized | p.11, line 175, Methods: Analysis & p.13-14, Results: Table 2 |  |
|  |  | (*c*) If relevant, consider translating estimates of relative risk into absolute risk for a meaningful time period | Not applicable |  |

Continued on next page

| Other analyses | 17 | Report other analyses done—eg analyses of subgroups and interactions, and sensitivity analyses | Not applicable |  |
| --- | --- | --- | --- | --- |
| Discussion | | | | |
| Key results | 18 | Summarise key results with reference to study objectives | p.18, line 254-265, Discussion |  |
| Limitations | 19 | Discuss limitations of the study, taking into account sources of potential bias or imprecision. Discuss both direction and magnitude of any potential bias | p.22, line 346-362, Discussion |  |
| Interpretation | 20 | Give a cautious overall interpretation of results considering objectives, limitations, multiplicity of analyses, results from similar studies, and other relevant evidence | p.18-21, line 266-345, Discussion |  |
| Generalisability | 21 | Discuss the generalisability (external validity) of the study results | p.22, line 359-362, Discussion |  |
| Other information | |  | | |
| Funding | 22 | Give the source of funding and the role of the funders for the present study and, if applicable, for the original study on which the present article is based | p.25, line 412-420, Funding information |  |

*Give information separately for cases and controls in case-control studies and, if applicable, for exposed and unexposed groups in cohort and cross-sectional studies.

**Note:** An Explanation and Elaboration article discusses each checklist item and gives methodological background and published examples of transparent reporting. The STROBE checklist is best used in conjunction with this article (freely available on the Web sites of PLoS Medicine at http://www.plosmedicine.org/, Annals of Internal Medicine at http://www.annals.org/, and Epidemiology at http://www.epidem.com/). Information on the STROBE Initiative is available at www.strobe-statement.org.
