# Supplementary material for: (De)coding perinatal deaths: applying the International Classification of Diseases to perinatal mortality to a perinatal e-registry from 16 hospitals in Benin, Malawi, Tanzania, and Uganda
Source: Glob Health Action. 2026 Aug 4;19(1):2709890. doi: 10.1080/16549716.2026.2709890 (PMC13439779; doi:10.1080/16549716.2026.2709890)
Supplement: Supplementary file 2 [file ZGHA_A_2709890_SM5805.docx]

Supplementary table 1. ALERT questionnaire

Supplementary table 2. Application of the ICD-PM to registry data

| Supplementary table 2 Application of the ICD-PM classification system to ALERT e-registry data from 16 hospitals in Benin, Malawi, Tanzania, and Uganda | | | | | |
| --- | --- | --- | --- | --- | --- |
| ICD-PM group | Level of detail | ALERT questionnaire | | | |
|  |  | Number of variables | Section | Question | Definition |
| Antepartum stillbirths | | | | |  |
| A1 - Congenital malformations & chromosomal abnormalities | Not detailed | 1 | Baby morbidity | Malformations | Structural defect to the body due to abnormal embryonic or fetal development (e.g. cleft lip/palate, spina bifida, club foot). |
| A2 - Infection | Not detailed | 1 | Baby morbidity | Neonatal infection | Infection of the neonate which is acquired before birth or in the first four weeks of life. Neonatal infections may be contracted by mother to child transmission, in the birth canal during childbirth, or after birth. |
| A3 - Hypoxia | Partially detailed | 3 | Indication for operative birth | Fetal distress | Signs before and during childbirth indicating that the fetus is not well. |
|  |  |  | Maternal complications | Fetal distress | See above |
|  |  |  | Baby morbidity | Asphyxia/hypo-ischemic insult | Asphyxia occurs when the body is deprived of oxygen. A hypo-ischemic insult is a brain injury that is caused by oxygen deprivation to the brain. This typically occurs in labor. |
| A4 - Other specified disorders | No data | - | - | - | - |
| A5 - Disorders related to fetal growth | Partially detailed | 3 | Antenatal care indicators | Gestational age | Number of completed weeks |
|  |  |  | Labor and birth | Birthweight | Measured in grams |
|  |  |  | Maternal complications | Fetal retardation/small for GA | Smaller in size than normal for gestational age, most commonly defined as a weight below the 10th percentile for the gestational age. |
| A6 - Unspecified cause | Not applicable | - | - | - | - |
| Intrapartum stillbirths | | | | |  |
| I1- Congenital malformations & chromosomal abnormalities | Not detailed | 1 | Baby morbidity | Malformations | See above |
| I2 - Birth trauma | No data | - | - | - | - |
| I3 - Acute intrapartum event | Partially detailed | 5 | Labor and birth | Fetal heartbeat abnormality at any time during 1^st^ or 2^nd^ stage of labor | Any abnormality in the heart rate of the baby. These can include tachycardia or bradycardia. The normal heart rate for a fetus is between 110 and 160 beats per minute. |
|  |  |  | Indication for operative birth | Fetal distress | See above |
|  |  |  | Maternal complications | Fetal distress | See above |
|  |  |  | Baby morbidity | Asphyxia/hypo-ischemic insult | See above |
| I4 - Infection | Not detailed | 1 | Baby morbidity | Neonatal infection | See above |
| I5 - Other specified disorders | No data | - | - | - | - |
| I6 - Disorders related to fetal growth | Partially detailed | 4 | Antenatal care indicators | Gestational age | See above |
|  |  |  | Labor and birth | Birthweight | See above |
|  |  |  | Maternal complications | Fetal retardation/small for GA | See above |
|  |  |  | Baby morbidity | Preterm birth | Baby is born before 37 completed gestational weeks |
| I7 - Unspecified cause | Not applicable | - | - | - | - |
| Neonatal death | | | | |  |
| N1 - Congenital malformations & chromosomal abnormalities | Not detailed | 1 | Baby morbidity | Malformations | See above |
| N2 - Disorders related to fetal growth | Partially detailed | 3 | Antenatal care indicators | Gestational age | See above |
|  |  |  | Labor and birth | Birthweight | See above |
|  |  |  | Maternal complications | Fetal retardation/small for GA | See above |
| N3 - Birth trauma | No data | - | - | - | - |
| N4 - Complications of intrapartum events | Partially detailed | 7 | Labor and birth | Fetal heartbeat abnormality at any time during 1st or 2nd stage of labor | See above |
|  |  |  |  | Any resuscitation, stimulation/ suction | Interventions given immediately after birth to help a newborn start breathing, clear the airway, or support circulation. |
|  |  |  |  | Apgar score at 5 minutes | Assessment of a newborn’s health 5 minutes after birth, based on breathing, heart rate, muscle tone, reflexes, and skin color. |
|  |  |  | Indication for operative birth | Fetal distress | See above |
|  |  |  | Maternal complications | Fetal distress | See above |
|  |  |  | Baby morbidity | Asphyxia/hypo-ischemic insult | See above |
| N5 – Convulsions and disorders of cerebral status | Detailed | 2 | Maternal complications | Seizures any/ever | Uncontrollable shaking of the whole body, or a part of the body, periods of unconsciousness, and bladder incontinence may occur |
|  |  |  | Baby interventions | MG-sulfate for neuroprotection of neonate | Baby received Magnesium Sulphate for neuroprotection |
| N6 - Infection | Not detailed | 2 | Baby morbidity | Neonatal infection | See above |
|  |  |  | Baby interventions | Received antibiotics | Baby received prophylactic or curative antibiotics. |
| N7 - Respiratory and cardiovascular disorders | Partially detailed | 3 | Resuscitation | Bag & mask  Chest compressions  CPAP | Any of these interventions were conducted |
| N8 - Other neonatal conditions | No data | - | - | - | - |
| N9 – Low birthweight and prematurity | Partially detailed | 3 | Labor and birth | Birthweight | See above |
|  |  |  | Maternal complications | Fetal retardation/small for GA | See above |
|  |  |  | Baby morbidity | Preterm birth | See above |
| N10 – Miscellaneous | No data | - | - | - | - |
| N11 – Unspecified cause | No data | - | - | - | - |
| Maternal conditions | | | | |  |
| M1 - Complications of placenta, cord, and membranes | Partially detailed | 6 | Antenatal complications | Cord complications | Umbilical cord drops between the fetal presenting part and the cervix into the vagina prior to or during delivery of the baby. |
|  |  |  | Indication for operative birth | Antepartum hemorrhage | Vaginal bleeding after or at 28 weeks of gestation or greater that is unrelated to labor and delivery. Includes placenta previa and placenta abruptio. |
|  |  |  |  | Cord complications | See above |
|  |  |  | Maternal complications | Antepartum hemorrhage | See above |
|  |  |  |  | Cord complications | See above |
|  |  |  |  | Chorioamnionitis | Fever (˃37.8°C) and at least two of the following: maternal HR >100, maternal WBC)> 15,000, uterine tenderness, fetal tachycardia > 160 and foul-smelling amniotic fluid. |
| M2 - Maternal complications of pregnancy | Partially detailed | 7 | Antenatal complications | Pre-labor rupture of membranes | Rupture of the fetal membranes prior to the onset of regular uterine contractions  Clinical criteria: Suggestive history (draining of clear fluid from the vagina in a sudden gush or trickling down the legs) AND sterile speculum examination that demonstrated oozing of fluid from the cervix either spontaneously or after a Valsalva maneuver (cough or strain). |
|  |  |  |  | Multiple pregnancy | Pregnancy with more than one fetus. |
|  |  |  | Indication for induction of labor | Pre-labor rupture of membranes | See above |
|  |  |  | Indication for operative birth | Pre-labor rupture of membranes | See above |
|  |  |  |  | Multiple pregnancy | See above |
|  |  |  | Labor and birth | Number of babies | Number of delivered babies irrespective of baby’s outcome. |
|  |  |  | Status of mother (at discharge or latest at day 7) | Died | Maternal outcome at discharge or day 7. |
| M3 - Other complications of labor and delivery | Partially detailed | 5 | Labor and birth | Mode of birth  -CS  -Vacuum extraction  -Forceps  -Assisted breech | CS: planned or unplanned surgical procedure to deliver a baby through incisions in the mother’s abdomen and uterus.  Vacuum extraction: Assisted vaginal delivery using a suction device (vacuum) attached to the baby’s head.  Forceps: Assisted vaginal delivery by using forceps to extract the fetus from the vagina, with or without the assistance of maternal pushing  Assisted breech: fetus descends spontaneously up to umbilicus then the rest is extracted using additional maneuver |
|  |  |  |  | Presentation of baby (breech or transverse) | Breech: the fetus’ presenting part is the buttocks and/or feet  Transverse: the fetus lies in a horizontal position across the maternal pelvis, resulting in the shoulder or arm presenting first |
|  |  |  |  | Precipitate delivery (variable calculated using start of labor and time of birth) | Baby is born within less than three hours from the start of labor |
|  |  |  | Indication of operative birth | Breech or other malpresentation | Breech: the fetus’ presenting part is the buttocks and/or feet |
| M4 - Maternal medical and surgical conditions | Partially detailed | 18 | Antenatal care indicators | Syphilis results | Positive results of laboratory testing |
|  |  |  |  | HIV results | Positive results of laboratory testing |
|  |  |  | Antenatal complications | Hypertensive disorders | Group of high blood pressure disorders that include preeclampsia, eclampsia, gestational hypertension, and chronic hypertension |
|  |  |  |  | Cardiac/renal diseases | Disorders of the heart/ kidneys |
|  |  |  |  | Severe anemia | Hemoglobin less than 7g/l |
|  |  |  |  | Malaria | Clinical and/or microscopic diagnosis. |
|  |  |  |  | Urinary tract infection | Asymptomatic bacteriuria, lower tract (acute cystitis) or upper tract (acute pyelonephritis) infection. |
|  |  |  |  | Gestational diabetes | Onset or first recognition of abnormal glucose tolerance during pregnancy |
|  |  |  |  | Diabetes mellitus | Chronic metabolic condition characterized by abnormal glucose tolerance |
|  |  |  | Indication for labor induction | Hypertensive disorders | See above |
|  |  |  | Indication for operative birth | Hypertensive disorders | See above |
|  |  |  | Maternal complication | Hypertensive disorders | See above |
|  |  |  |  | Malaria | See above |
|  |  |  |  | Puerperal sepsis | Infection of the genital tract occurring at any time between the onset of rupture of membranes or labor and the 42nd day postpartum in which two or more of the following are present: pelvic pain, fever, abnormal vaginal discharge, abnormal smell/foul odor discharge or delay in uterine involution. |
|  |  |  |  | Other infections (non-puerperal) | Maternal infections unrelated to the puerperal period, including infections acquired before pregnancy, during pregnancy, or from other sources. |
|  |  |  |  | Urinary tract infection | See above |
|  |  |  |  | Gestational diabetes | See above |
|  |  |  | Perinatal intervention | Anti-hypertensive treatment | Mother received medication to lower blood pressure. |
|  |  |  |  | Curative ATB | Mother received antibiotics to cure a confirmed infection. |
| M5 - No maternal condition | Not applicable | - | - | - | - |

Supplementary table 3. Main characteristics of study population

| Supplementary table 3 Main maternal, obstetric, and fetal characteristics of 143,105 births across 16 hospitals in Benin, Malawi, Tanzania, and Uganda | | | | | | | | | | |
| --- | --- | --- | --- | --- | --- | --- | --- | --- | --- | --- |
|  | Benin  n= 31,933 | | Malawi  n= 50,541 | | Tanzania  n= 23,267 | | Uganda  n= 37,364 | | Pooled  n= 143,105 | |
|  | n | % | n | % | n | % | n | % | n | % |
| Maternal age (years) | | | | | | | | | | |
| <20 | 3,121 | 9.8 | 16,080 | 31.8 | 4,606 | 19.8 | 6,544 | 17.5 | 30,351 | 21.2 |
| 20-29 | 17,128 | 53.6 | 24,247 | 48.0 | 10,917 | 46.9 | 21,850 | 58.5 | 74,142 | 51.8 |
| 30-39 | 10,659 | 33.4 | 9,091 | 18.0 | 6,509 | 28.0 | 8,468 | 22.7 | 34,727 | 24.3 |
| ≥40 | 1,009 | 3.2 | 1,023 | 2.0 | 1,235 | 5.3 | 497 | 1.3 | 3,764 | 2.6 |
| Missing | 16 | 0.1 | 100 | 0.2 | 0 | 0.0 | 5 | 0.0 | 121 | 0.1 |
| Parity at index pregnancy | | | | | | | | | | |
| 0 | 9,290 | 29.1 | 23,612 | 46.7 | 8,616 | 37.0 | 13,250 | 35.5 | 54,768 | 38.3 |
| 1-3 births | 16,120 | 50.5 | 22,404 | 44.3 | 12,659 | 54.4 | 18,494 | 49.5 | 69,677 | 48.7 |
| ≥4 births | 5,975 | 18.7 | 4,459 | 8.8 | 1,912 | 8.2 | 5,553 | 14.9 | 17,899 | 12.5 |
| Missing | 548 | 1.7 | 66 | 0.1 | 80 | 0.3 | 67 | 0.2 | 761 | 0.5 |
| Poor outcome in immediate previous pregnancy | | | | | | | | | | |
| No | 19,198 | 60.1 | 24,526 | 48.5 | 13,517 | 58.1 | 20,755 | 55.5 | 77,996 | 54.5 |
| Yes^a^ | 4,023 | 12.6 | 3,502 | 6.9 | 1,552 | 6.7 | 3,483 | 9.3 | 12,560 | 8.8 |
| No previous pregnancy | 8,152 | 25.5 | 22,403 | 44.3 | 8,114 | 34.9 | 12,117 | 32.4 | 50,786 | 35.5 |
| Missing | 560 | 1.8 | 110 | 0.2 | 84 | 0.4 | 1,009 | 2.7 | 1,763 | 1.2 |
| Number of antenatal care visits for index pregnancy | | | | | | | | | | |
| None | 256 | 0.8 | 1,668 | 3.3 | 19 | 0.1 | 188 | 0.5 | 2,131 | 1.5 |
| 1-3 | 8,609 | 27.0 | 22,828 | 45.2 | 1,940 | 8.3 | 15,758 | 42.2 | 49,135 | 34.3 |
| 4-7 | 20,718 | 64.9 | 25,103 | 49.7 | 20,200 | 86.8 | 20,515 | 54.9 | 86,536 | 60.5 |
| ≥8 | 2,131 | 6.7 | 598 | 1.2 | 1,105 | 4.7 | 577 | 1.5 | 4,411 | 3.1 |
| Missing | 219 | 0.7 | 344 | 0.7 | 3 | 0.0 | 326 | 0.9 | 892 | 0.6 |
| Antenatal complications in index pregnancy^b^ | 10,582 | 33.2 | 2,717 | 5.4 | 1,946 | 8.4 | 4,204 | 11.3 | 19,449 | 13.6 |
| Multiple pregnancy | 4,853 | 15.2 | 1,718 | 3.4 | 914 | 3.9 | 2,308 | 6.2 | 9,793 | 6.8 |
| Fetal heartbeat at admission | | | | | | | | | | |
| Positive | 26,049 | 81.6 | 47,481 | 93.9 | 21,094 | 90.7 | 33,369 | 89.3 | 127993 | 89.4 |
| Negative | 1,930 | 6.0 | 678 | 1.3 | 456 | 2.0 | 1,467 | 3.9 | 4,531 | 3.2 |
| No information | 3,954 | 12.4 | 2,382 | 4.7 | 1,717 | 7.4 | 2,528 | 6.8 | 10,581 | 7.4 |
| Referred from another health facility | 16,496 | 51.7 | 4,620 | 9.1 | 1,176 | 5.1 | 5,229 | 14.0 | 27,521 | 19.2 |
| Mode of birth | | | | | | | | | | |
| Vaginal | 17,053 | 53.4 | 40,721 | 80.6 | 16,174 | 69.5 | 26,745 | 71.6 | 100693 | 70.4 |
| Cesarean Section | 14,439 | 45.2 | 8,880 | 17.6 | 6,922 | 29.8 | 10,470 | 28.0 | 40,711 | 28.4 |
| Assisted breech | 225 | 0.7 | 527 | 1.0 | 98 | 0.4 | 136 | 0.4 | 986 | 0.7 |
| Operative vaginal | 216 | 0.7 | 413 | 0.8 | 73 | 0.3 | 13 | 0.0 | 715 | 0.5 |
| Gestational age (weeks) | | | | | | | | | | |
| <32 | 899 | 2.8 | 481 | 1.0 | 264 | 1.1 | 567 | 1.5 | 2,211 | 1.5 |
| 32-36 | 6,134 | 19.2 | 4,789 | 9.5 | 2,900 | 12.5 | 3,509 | 9.4 | 17,332 | 12.1 |
| 37-41 | 23,609 | 73.9 | 44,076 | 87.2 | 18,642 | 80.1 | 29,772 | 79.7 | 116099 | 81.1 |
| ≥42 | 1,044 | 3.3 | 682 | 1.3 | 1,419 | 6.1 | 1,751 | 4.7 | 4,896 | 3.4 |
| Missing | 247 | 0.8 | 513 | 1.0 | 42 | 0.2 | 1,765 | 4.7 | 2,567 | 1.8 |
| Birthweight (grams) | | | | | | | | | | |
| <1500 | 903 | 2.8 | 468 | 0.9 | 262 | 1.1 | 435 | 1.2 | 2,068 | 1.4 |
| 1500-2499 | 7,339 | 23.0 | 6,884 | 13.6 | 2,818 | 12.1 | 3,551 | 9.5 | 20,592 | 14.4 |
| 2500-3999 | 23,175 | 72.6 | 42,296 | 83.7 | 19,818 | 85.2 | 31,044 | 83.1 | 116333 | 81.3 |
| ≥4000 | 409 | 1.3 | 765 | 1.5 | 363 | 1.6 | 2,206 | 5.9 | 3,743 | 2.6 |
| Missing | 107 | 0.3 | 128 | 0.3 | 6 | 0.0 | 128 | 0.3 | 369 | 0.3 |
| Baby's sex |  |  |  |  |  |  |  |  |  |  |
| Female | 15,176 | 47.5 | 24,398 | 48.3 | 11,529 | 49.6 | 18,760 | 50.2 | 69,863 | 48.8 |
| Male | 16,740 | 52.4 | 26,131 | 51.7 | 11,733 | 50.4 | 18,598 | 49.8 | 73,202 | 51.2 |
| Missing | 17 | 0.1 | 12 | 0.0 | 5 | 0.0 | 6 | 0.0 | 40 | 0.0 |
| a Includes miscarriage, stillbirth, and neonatal death  b Includes: hypertensive disorder, preterm labor, pre-labor rupture of membranes, cardiac/renal diseases, gestational diabetes, diabetes mellitus, malaria, and severe anemia | | | | | | | | | | |

Supplementary table 4.

| Supplementary Table 4 Sensitivity analysis using FHR at admission to determine time of death | | | | | | |
| --- | --- | --- | --- | --- | --- | --- |
|  | Classification based on physical appearance | | | | | |
|  | Macerated stillbirths | | Fresh stillbirths | | Total | |
|  | n | % | n | % | n | % |
| Classification based on FHR at admission |  |  |  |  |  |  |
| Antepartum stillbirth | 1,780 | 84.8 | 1,585 | 62.9 | 3,365 | 72.9 |
| Intrapartum stillbirth | 318 | 15.2 | 933 | 37.1 | 1,251 | 27.1 |

Supplementary table 5. Stillbirth and very early neonatal mortality rates per hospital

| Supplementary table 5 Stillbirth and very early neonatal mortality rates per hospital | | |
| --- | --- | --- |
|  | Stillbirth rate  (per 1,000 births) | Very early neonatal mortality rate  (per 1,000 live births) |
| Benin |  |  |
| De Zone Come-Grand Popo Houeyogbe Bop | 51.9 | 7.8 |
| Centre et Dépt Zou-Collines | 85.6 | 7.9 |
| Centre National Hubert Maga | 65.6 | 12.5 |
| Policlinique Bon Samaritan | 30.7 | 3.5 |
| Malawi |  |  |
| Mchinji | 23.9 | 9.6 |
| Ntcheu | 12.4 | 3.8 |
| Mitundu | 21.1 | 5.7 |
| St. Gabriels | 19.3 | 8.0 |
| Tanzania |  |  |
| Ndanda | 31.8 | 5.2 |
| Mkomaindo | 14.4 | 6.2 |
| Nachingwea | 13.3 | 2.2 |
| Newala | 7.3 | 3.1 |
| Uganda |  |  |
| Jinja Regional | 64.9 | 7.3 |
| Kamuli | 72.8 | 14.0 |
| Iganga | 35.5 | 11.8 |
| Bugiri | 35.7 | 6.4 |

Supplementary table 6. Classification of 6,338 stillbirths and early neonatal deaths according to cause of death and associated maternal condition using the ICD-PM reported by country

| Supplementary table 6 Classification of 6,338 stillbirths and early neonatal deaths according to cause of death and associated maternal condition using the ICD-PM reported by country | | | | | | |
| --- | --- | --- | --- | --- | --- | --- |
| Benin | | | | | | |
|  | Maternal conditions* | | | | | |
|  | M1 | M2 | M3 | M4 | M5 | Total  (%) |
| Antepartum death | | | | | | |
| A1: Congenital malformations, deformations and chromosomal abnormalities | 0 | 6 | 9 | 3 | 3 | 21  (2.3) |
| A2: Infection | 3 | 7 | 4 | 3 | 0 | 17  (1.8) |
| A3: Antepartum hypoxia | 8 | 12 | 10 | 4 | 2 | 36  (3.9) |
| A4: Other specified antepartum disorders | ND | ND | ND | ND | ND | ND |
| A5: Disorders related to fetal growth | 6 | 3 | 15 | 12 | 4 | 40  (4.3) |
| A6: Fetal death of unspecified cause | 101 | 165 | 277 | 227 | 45 | 815  (87.7) |
| Total (%) | 118  (12.7) | 193  (20.8) | 315  (33.9) | 249  (26.8) | 54  (5.8) | 929  (100.0) |
| Intrapartum death | | | | | | |
| I1: Congenital malformations, deformations and chromosomal abnormalities | 3 | 9 | 16 | 9 | 2 | 39  (3.1) |
| I2: Birth trauma | ND | ND | ND | ND | ND | ND |
| I3: Acute intrapartum event | 29 | 45 | 133 | 36 | 3 | 246  (19.7) |
| I4: Infection | 0 | 3 | 0 | 0 | 0 | 3  (0.2) |
| I5: Other specified antepartum disorders | ND | ND | ND | ND | ND | ND |
| I6: Disorders related to fetal growth | 167 | 97 | 162 | 63 | 10 | 499  (39.9) |
| I7: Intrapartum death of unspecified cause | 106 | 64 | 219 | 67 | 8 | 464  (37.1) |
| Total (%) | 305  (24.4) | 218  (17.4) | 530  (42.4) | 175  (14.0) | 23  (1.8) | 1,251  (100.0) |
| Neonatal death | | | | | | |
| N1: Congenital malformations, deformations and chromosomal abnormalities | 2 | 5 | 8 | 0 | 2 | 17  (6.7) |
| N2: Disorders related to fetal growth | 2 | 1 | 10 | 1 | 2 | 16  (6.3) |
| N3: Birth trauma | ND | ND | ND | ND | ND | ND |
| N4: Complications of intrapartum events | 28 | 48 | 87 | 30 | 7 | 200  (78.4) |
| N5: Convulsions and disorders of cerebral status | 0 | 1 | 1 | 1 | 0 | 3  (1.2) |
| N6: Infection | 0 | 0 | 0 | 0 | 0 | 0 |
| N7: Respiratory and cardiovascular disorders | 0 | 0 | 0 | 0 | 0 | 0 |
| N8: Other neonatal conditions | ND | ND | ND | ND | ND | ND |
| N9: Low birth weight and prematurity | 1 | 12 | 6 | 0 | 0 | 19  (7.5) |
| N10: Miscellaneous | ND | ND | ND | ND | ND | ND |
| N11: Neonatal death of unspecified cause | ND | ND | ND | ND | ND | ND |
| Total (%) | 33  (12.9) | 67  (26.3) | 112  (43.9) | 32  (12.5) | 11  (4.3) | 255  (100.0) |

| Malawi | | | | | | |
| --- | --- | --- | --- | --- | --- | --- |
|  | Maternal conditions* | | | | | |
|  | M1 | M2 | M3 | M4 | M5 | Total (%) |
| Antepartum death | | | | | | |
| A1: Congenital malformations, deformations and chromosomal abnormalities | 0 | 0 | 0 | 0 | 1 | 1  (0.2) |
| A2: Infection | 0 | 0 | 1 | 0 | 0 | 1  (0.2) |
| A3: Antepartum hypoxia | 0 | 1 | 5 | 0 | 3 | 9  (1.8) |
| A4: Other specified antepartum disorders | ND | ND | ND | ND | ND | ND |
| A5: Disorders related to fetal growth | 0 | 3 | 3 | 3 | 10 | 19  (3.8) |
| A6: Fetal death of unspecified cause | 12 | 61 | 62 | 83 | 250 | 468  (94.0) |
| Total (%) | 12  (2.4) | 65  (13.1) | 71  (14.3) | 86  (17.3) | 264  (53.0) | 498  (100.0) |
| Intrapartum death | | | | | | |
| I1: Congenital malformations, deformations and chromosomal abnormalities | 0 | 2 | 1 | 0 | 5 | 8  (1.7) |
| I2: Birth trauma | ND | ND | ND | ND | ND | ND |
| I3: Acute intrapartum event | 16 | 17 | 74 | 12 | 80 | 199  (41.7) |
| I4: Infection | 0 | 0 | 0 | 0 | 0 | 0 |
| I5: Other specified antepartum disorders | ND | ND | ND | ND | ND | ND |
| I6: Disorders related to fetal growth | 18 | 27 | 26 | 11 | 43 | 125  (26.2) |
| I7: Intrapartum death of unspecified cause | 16 | 9 | 45 | 4 | 71 | 145  (30.4) |
| Total (%) | 50  (10.5) | 55  (11.5) | 146  (30.6) | 27  (5.7) | 199  (41.7) | 477  (100.0) |
| Neonatal death | | | | | | |
| N1: Congenital malformations, deformations and chromosomal abnormalities | 0 | 2 | 7 | 0 | 3 | 12  (3.5) |
| N2: Disorders related to fetal growth | 0 | 3 | 4 | 0 | 2 | 9  (2.6) |
| N3: Birth trauma | ND | ND | ND | ND | ND | ND |
| N4: Complications of intrapartum events | 12 | 24 | 84 | 26 | 167 | 313  (90.7) |
| N5: Convulsions and disorders of cerebral status | 0 | 0 | 0 | 0 | 0 | 0 |
| N6: Infection | 0 | 0 | 0 | 0 | 0 | 0 |
| N7: Respiratory and cardiovascular disorders | 0 | 0 | 0 | 0 | 0 | 0 |
| N8: Other neonatal conditions | ND | ND | ND | ND | ND | ND |
| N9: Low birth weight and prematurity | 2 | 6 | 0 | 1 | 2 | 11  (3.2) |
| N10: Miscellaneous | ND | ND | ND | ND | ND | ND |
| N11: Neonatal death of unspecified cause | ND | ND | ND | ND | ND | ND |
| Total (%) | 14  (4.1) | 35  (10.1) | 95  (27.5) | 27  (7.8) | 174  (50.4) | 345  (100.0) |

| Tanzania | | | | | | |
| --- | --- | --- | --- | --- | --- | --- |
|  | Maternal conditions* | | | | | |
|  | M1 | M2 | M3 | M4 | M5 | Total (%) |
| Antepartum death | | | | | | |
| A1: Congenital malformations, deformations and chromosomal abnormalities | 1 | 1 | 2 | 0 | 0 | 4  (2.0) |
| A2: Infection | 0 | 0 | 0 | 0 | 0 | 0 |
| A3: Antepartum hypoxia | 0 | 1 | 2 | 0 | 1 | 4  (2.0) |
| A4: Other specified antepartum disorders | ND | ND | ND | ND | ND | ND |
| A5: Disorders related to fetal growth | 3 | 0 | 4 | 3 | 4 | 14  (6.9) |
| A6: Fetal death of unspecified cause | 8 | 30 | 45 | 42 | 57 | 182  (89.2) |
| Total (%) | 12  (5.9) | 32  (15.7) | 53  (26.0) | 45  (22.1) | 62  (30.4) | 204  (100.0) |
| Intrapartum death | | | | | | |
| I1: Congenital malformations, deformations and chromosomal abnormalities | 0 | 0 | 3 | 0 | 2 | 5  (3.2) |
| I2: Birth trauma | ND | ND | ND | ND | ND | ND |
| I3: Acute intrapartum event | 10 | 15 | 21 | 3 | 8 | 57  (36.3) |
| I4: Infection | 0 | 0 | 0 | 0 | 0 | 0 |
| I5: Other specified antepartum disorders | ND | ND | ND | ND | ND | ND |
| I6: Disorders related to fetal growth | 15 | 18 | 12 | 11 | 7 | 63  (40.1) |
| I7: Intrapartum death of unspecified cause | 7 | 4 | 11 | 5 | 5 | 32  (20.4) |
| Total (%) | 32  (20.4) | 37  (23.6) | 47  (29.9) | 19  (12.1) | 22  (14.0) | 157  (100.0) |
| Neonatal death | | | | | | |
| N1: Congenital malformations, deformations and chromosomal abnormalities | 0 | 1 | 1 | 1 | 2 | 5  (5.4) |
| N2: Disorders related to fetal growth | 0 | 0 | 1 | 0 | 4 | 5  (5.4) |
| N3: Birth trauma | ND | ND | ND | ND | ND | ND |
| N4: Complications of intrapartum events | 6 | 14 | 20 | 5 | 33 | 78  (83.9) |
| N5: Convulsions and disorders of cerebral status | 0 | 0 | 0 | 0 | 0 | 0 |
| N6: Infection | 0 | 0 | 0 | 0 | 0 | 0 |
| N7: Respiratory and cardiovascular disorders | 0 | 0 | 0 | 0 | 0 | 0 |
| N8: Other neonatal conditions | ND | ND | ND | ND | ND | ND |
| N9: Low birth weight and prematurity | 0 | 1 | 0 | 2 | 2 | 5  (5.4) |
| N10: Miscellaneous | ND | ND | ND | ND | ND | ND |
| N11: Neonatal death of unspecified cause | ND | ND | ND | ND | ND | ND |
| Total (%) | 6  (6.5) | 16  (17.2) | 22  (23.7) | 8  (8.6) | 41  (44.1) | 93  (100.0) |

| Uganda | | | | | | |
| --- | --- | --- | --- | --- | --- | --- |
|  | Maternal conditions* | | | | | |
|  | M1 | M2 | M3 | M4 | M5 | Total (%) |
| Antepartum death | | | | | | |
| A1: Congenital malformations, deformations and chromosomal abnormalities | 1 | 1 | 1 | 3 | 0 | 6  (0.8) |
| A2: Infection | 0 | 0 | 0 | 0 | 0 | 0 |
| A3: Antepartum hypoxia | 2 | 2 | 5 | 2 | 0 | 11  (1.6) |
| A4: Other specified antepartum disorders | ND | ND | ND | ND | ND | ND |
| A5: Disorders related to fetal growth | 2 | 1 | 9 | 19 | 11 | 42  (5.9) |
| A6: Fetal death of unspecified cause | 44 | 68 | 128 | 243 | 167 | 650  (91.7) |
| Total (%) | 49  (6.9) | 72  (10.2) | 143  (20.2) | 267  (37.7) | 178  (25.1) | 709  (100.0) |
| Intrapartum death | | | | | | |
| I1: Congenital malformations, deformations and chromosomal abnormalities | 1 | 2 | 9 | 6 | 1 | 19  (1.8) |
| I2: Birth trauma | ND | ND | ND | ND | ND | ND |
| I3: Acute intrapartum event | 28 | 17 | 88 | 20 | 9 | 162  (15.4) |
| I4: Infection | 0 | 0 | 0 | 1 | 0 | 1  (0.1) |
| I5: Other specified antepartum disorders | ND | ND | ND | ND | ND | ND |
| I6: Disorders related to fetal growth | 99 | 63 | 83 | 135 | 51 | 431  (40.9) |
| I7: Intrapartum death of unspecified cause | 64 | 52 | 152 | 105 | 68 | 441  (41.8) |
| Total (%) | 192  (18.2) | 134  (12.7) | 332  (31.5) | 267  (25.3) | 129  (12.2) | 1,054  (100.0) |
| Neonatal death | | | | | | |
| N1: Congenital malformations, deformations and chromosomal abnormalities | 1 | 1 | 6 | 2 | 3 | 13  (3.8) |
| N2: Disorders related to fetal growth | 1 | 2 | 10 | 9 | 7 | 29  (8.4) |
| N3: Birth trauma | ND | ND | ND | ND | ND | ND |
| N4: Complications of intrapartum events | 29 | 34 | 104 | 47 | 62 | 276  (80.0) |
| N5: Convulsions and disorders of cerebral status | 0 | 0 | 1 | 0 | 0 | 1  (0.3) |
| N6: Infection | 0 | 0 | 0 | 0 | 0 | 0 |
| N7: Respiratory and cardiovascular disorders | 0 | 0 | 0 | 0 | 0 | 0 |
| N8: Other neonatal conditions | ND | ND | ND | ND | ND | ND |
| N9: Low birth weight and prematurity | 2 | 15 | 0 | 2 | 7 | 26  (7.5) |
| N10: Miscellaneous | ND | ND | ND | ND | ND | ND |
| N11: Neonatal death of unspecified cause | ND | ND | ND | ND | ND | ND |
| Total (%) | 33  (9.6) | 52  (15.1) | 121  (35.1) | 60  (17.4) | 79  (22.9) | 345  (100.0) |
| *M1: Complications of placenta, cord and membranes; M2: Maternal complications of pregnancy; M3: Other complications of labor and delivery; M4: Maternal medical and surgical conditions; M5: No maternal condition.  ND (no data): no suitable variable was available in the ALERT e-registry. | | | | | | |
